# Complete Remission of Plasmablastic Lymphoma With Daratumumab and a Mild Regimen of Cyclophosphamide, Doxorubicin, Vincristine, and Prednisolone (Mini-CHOP)

**DOI:** 10.7759/cureus.76733

**Published:** 2025-01-01

**Authors:** Samuel Z Davis, Wei Cui, Jordan Snyder, Forat Lutfi

**Affiliations:** 1 School of Medicine, University of Kansas Medical Center, Kansas City, USA; 2 Pathology and Laboratory Medicine, University of Kansas Medical Center, Kansas City, USA; 3 Pharmacology, The University of Kansas Cancer Center, Kansas City, USA; 4 Hematology, The University of Kansas Cancer Center, Kansas City, USA

**Keywords:** chemotherapy response, chop regimen, complete stable remission, daratumumab, elderly patients, plasmablastic large b-cell lymphoma

## Abstract

Plasmablastic lymphoma (PBL) is a rare and highly aggressive subtype of diffuse large B-cell lymphoma, characterized by a dismal prognosis. Due to its rarity and aggressive nature, no established standard of care exists for PBL. Treatment is primarily based on large B-cell lymphoma- or multiple myeloma-based chemotherapy regimens, and outcomes remain poor. We present an 84-year-old man with an extensive oncologic history who presented with PBL on his right foot which did not respond to subsequent radiation treatment. A mild regimen of cyclophosphamide, doxorubicin, vincristine, and prednisolone (mini-CHOP) normally utilized to treat large B-cell lymphoma was supplemented with the CD38-directed monoclonal antibody, daratumumab. This combination regimen was selected for the elderly patient due to his weakened condition. Upon six cycles of daratumumab-mini-CHOP, the patient achieved complete remission.

## Introduction

Plasmablastic lymphoma (PBL) is an aggressive form of diffuse large B-cell lymphoma that is more commonly seen in human immunodeficiency virus (HIV)-positive and immunocompromised patients [1-5]. It is exceedingly rare, comprising 2% of all HIV-related lymphomas, and difficult to diagnose because of its morphologic and immunophenotypic overlap with plasmablastic myeloma and lymphomas with plasmablastic differentiation [1-5].

Patients diagnosed with PBL experience abysmal prognoses. It has been shown that the median overall survival (OS) of HIV-positive PBL patients is 15 months with 25% of patients reaching a three-year OS [6]. Similarly, in HIV-negative PBL patients, the median survival was shown to be eight months with 10% of patients reaching a two-year OS [7]. Previous treatments have included chemotherapy regimens supplemented by monoclonal antibodies, hematopoietic stem cell transplantation, and immunotherapy, but no standard of care has been established because of its low incidence and poor survival rates. PBL has a similar immunophenotype and morphology to plasma cell myeloma which complicates therapeutic treatment [1]. It remains unclear whether patients would respond better to chemotherapy regimens targeting B-cell lymphoma or to regimens targeting myeloma or a combination of the two. Additionally, the rarity of PBL makes clinical trials difficult to assemble, further complicating the establishment of a standard of care.

The absence of an established standard of care makes designing an effective treatment plan for PBL particularly challenging, especially in elderly patients. Factors such as comorbidities, age-related changes in drug sensitivity, and the patient's ability to tolerate chemotherapy regimens further complicate treatment decisions. Additionally, balancing the potential therapeutic benefits of chemotherapy with the impact on the patient's quality of life adds another layer of complexity to managing PBL in older adults. Despite these obstacles, we present an 84-year-old man with a previous history of fibrosarcoma, basal cell carcinoma, and lymphoma of unknown type decades prior who achieved complete remission from PBL after six cycles of daratumumab and a mild regimen of cyclophosphamide, doxorubicin, vincristine, and prednisolone (mini-CHOP). The regimen consisted of the mini-CHOP regimen (rituximab held given PBL is CD20 negative) published by Peyrade et al. with mini-CHOP chemotherapy (cyclophosphamide: 400 mg/m^2^ day 1, doxorubicin: 25 mg/m^2^ day 1, vincristine: 1 mg day 1, and prednisolone: 40 mg/m^2^/day by oral route from days 1 to 5) plus daratumumab hyaluronidase, 1800 mg on days 1 and 8 of each 21-day cycle, followed by monthly daratumumab maintenance [8]. 

## Case presentation

**Figure 1:**
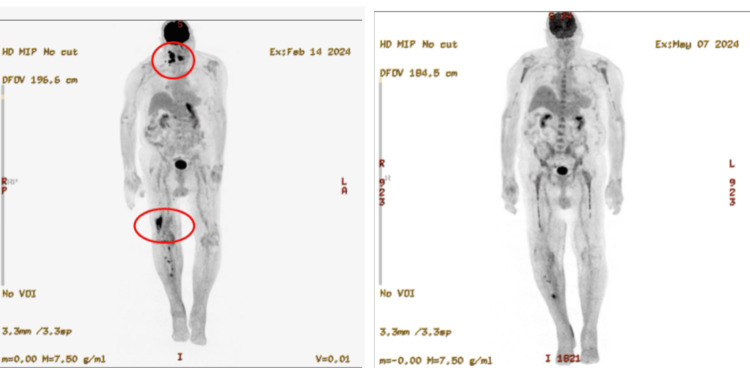
Frontal scout view showing the right cervical nodal conglomerate with an SUVmax of 12.7 on the left and complete resolution after therapy on the right SUV: standardized uptake value

**Figure 2:**
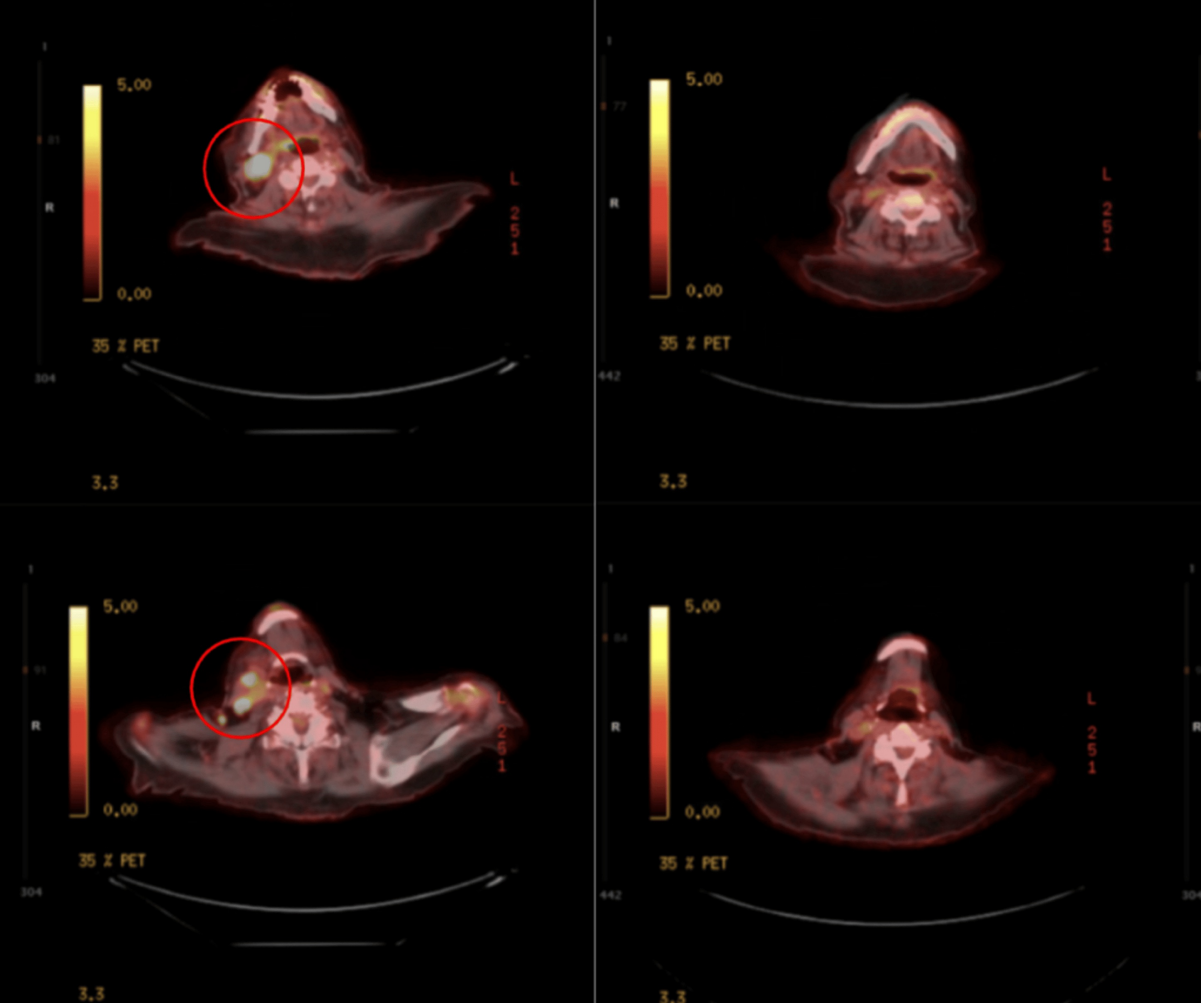
Axial views showing the cervical conglomerate on the left and complete resolution after therapy on the right

**Figure 3:**
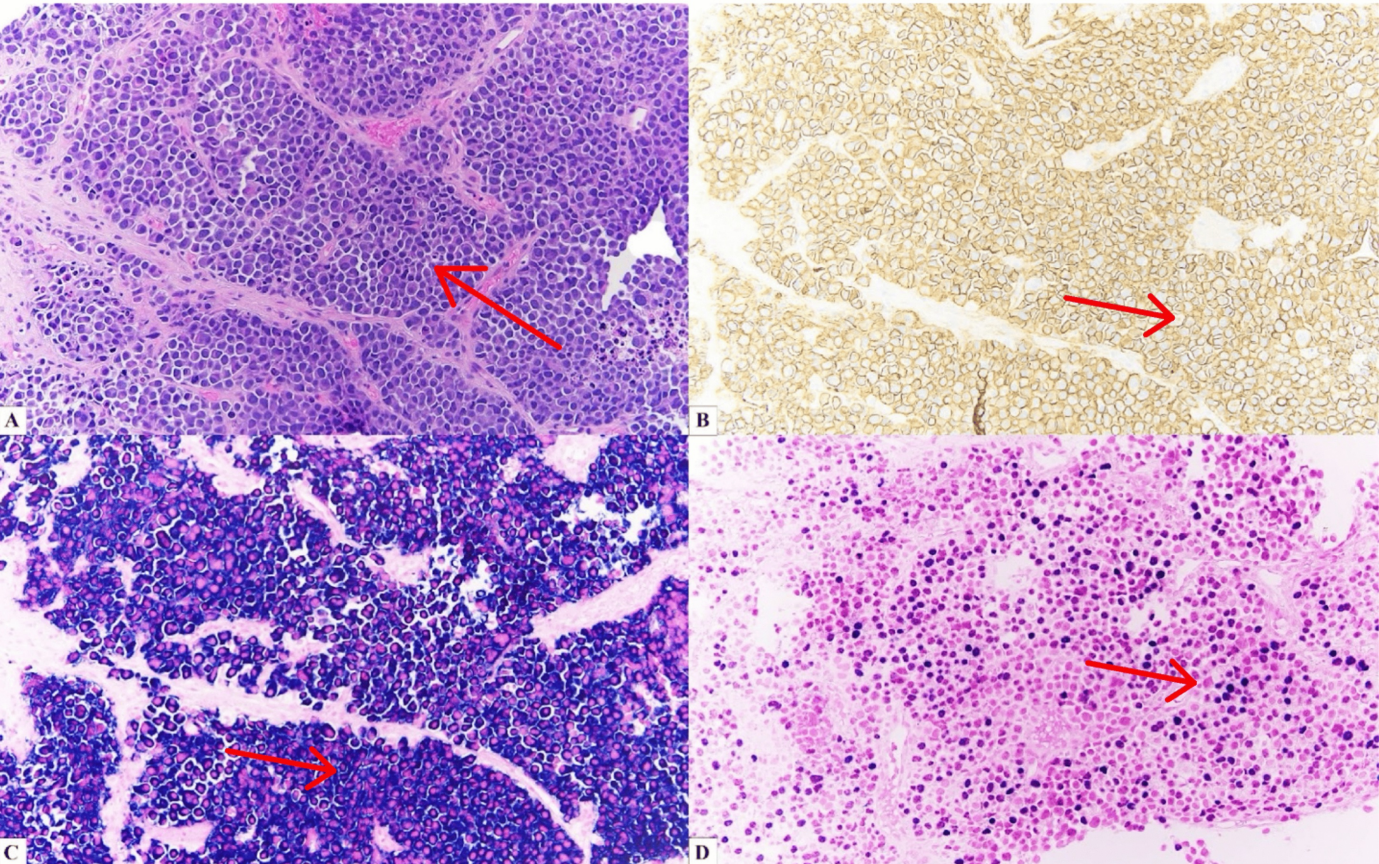
Morphologic and immunophenotypic features of plasmablastic lymphoma. (A) H&E of plasmablastic lymphoma (×200). (B) CD138 immunostain staining abundantly positive (×200). (C) Kappa in situ hybridization study demonstrating kappa restriction consistent with a monoclonal plasma cell population (×200). (D) EBV in situ hybridization study demonstrating EBV positivity (×200) H&E: hematoxylin and eosin; EBV: Epstein-Barr virus

We present an 84-year-old man with a previous history of fibrosarcoma, lymphoma of unknown type, squamous cell carcinoma, and basal cell carcinoma. The lymphoma of unknown type presented in his right proximal tibia and was treated with radiation therapy in 1977. The patient was diagnosed in 2023 with PBL following a skin biopsy of his right foot that showed an Epstein-Barr virus (EBV)-positive high-grade hematolymphoid neoplasm with plasmacytic differentiation. A positron emission tomography-computed tomography (PET/CT) scan showed no obvious lymphomatous involvement of the sural nerve and indicated that the lesion was confined to the right foot. Given these results, the patient was referred to radiation oncology where he was treated with 4500 centigray (cGy) in 25 fractions over the course of one month. Three months later, he unfortunately developed fluorodeoxyglucose (FGD) avid, right-cervical lymphadenopathy with a standardized uptake value (SUV)max of 12.7 (Figure 1 and Figure 2). A biopsy confirmed that the new mass was consistent with recurrent disease. The hematoxylin and eosin (H&E) section of the needle core biopsy showed complete effacement of the lymphoid architecture, replaced by sheets of lymphoma cells (Figure 3A). The lymphoma cells were large, with eccentric nuclei, prominent nucleoli, and abundant basophilic cytoplasm. Binucleation was present, along with frequent mitotic activity and areas of necrosis. The lymphoma cells were positive for CD138 (Figure 3B), MUM1, and C-MYC and negative for CD20, CD30, CD79a, PAX5, ALK-1, and HHV-8. In situ hybridization studies showed monoclonal kappa expression and positivity for EBV (Figure 3C, 3D). The Ki-67 proliferation index was greater than 95%. These results ruled out other plasma cell neoplasms, such as diffuse large B-cell lymphoma or myeloma, and confirmed that a new treatment plan was required. Radiation therapy was excluded because of the diffuse nature of his condition, and a regimen of bortezomib +/- etoposide, prednisone, vincristine, cyclophosphamide, and doxorubicin (EPOCH) was similarly excluded because of the patient's age and weakness. The final treatment plan consisted of six cycles of mini-CHOP with daratumumab, a CD38 targeting monoclonal antibody normally used for myeloma treatment, added in after the first cycle. While this regimen has never been reported to our knowledge, it was chosen given the known tolerance of mini-CHOP-based regimens in patients over 80 years old and the excellent tolerability of daratumumab. 

The treatment plan consisting of six cycles of daratumumab-mini-CHOP was well tolerated by the patient as an outpatient regimen and led to a complete remission of his lymphoma. The end-of-treatment PET/CT demonstrated a complete absence of both the right cervical conglomerate and the mass in the right lower extremity following the completion of treatment (Figure 1). Similarly, the axial views of the cervical tumor further demonstrate the effectiveness of the daratumumab-mini-CHOP regimen. Further consistent with disease response, serum EBV went from significantly elevated at 3605 international units/mL to detectable but unquantifiable by the end of treatment.

The patient was scheduled to receive subcutaneous maintenance doses of daratumumab every 28 days, but he only received one dose. He tolerated the regimen quite well with minimal side effects. His cause of death was due to decline rather than disease or therapy, and he was in hospice care at the time of his passing. 

## Discussion

Frontline treatment with a combination of bortezomib and dose-adjusted EPOCH (V-daEPOCH) has led to improved outcomes for patients with PBL [9-10]. However, many patients over the age of 60 experience febrile neutropenia, neuropathy, and infections while enduring this regimen [9]. In elderly patients, a mini-CHOP regimen has been shown to be more tolerable than more robust regimens like EPOCH [8]. Mini-CHOP supplemented with daratumumab was continually well tolerated by our patient, who achieved complete remission. 

Daratumumab, a CD38-targeting antibody, was added to our patient's chemotherapy regimen due to its proven tolerability and efficacy in treating hematologic tumors [11-13]. Tumors that express CD38, such as PBL and myelomas, are specifically targeted by daratumumab, which exerts its therapeutic effects through antibody-dependent cellular cytotoxicity, antibody-dependent cellular phagocytosis, complement-mediated lysis, and direct apoptosis [11]. The complete remission achieved by our patient adds to the growing body of evidence supporting the effectiveness of daratumumab in treating PBL. However, we acknowledge that further research needs to be conducted before the findings of this case study can be generalized to a larger patient population.

Case reports detailing spontaneous remission from PBL have suggested that lenalidomide-based therapy may also be an effective frontline treatment as an immunomodulating agent [14-17]. Usually used in myeloma treatment, lenalidomide is an immunomodulatory medication that downregulates a wide array of tumor machinery. Additionally, it has been shown to promote T-cell proliferation, activate NK cells, and inhibit pro-inflammatory cytokine production [18]. The effectiveness of lenalidomide in treating PBL may suggest that other myeloma-based therapies could show promising results in further clinical trials. 

Despite some improvements in chemotherapy treatment, prognoses for patients with PBL remain abysmal, but new therapeutic options are currently under investigation. The use of immune blockade therapy and kinase pathway inhibition have shown some efficacy in recent years [18-19]. Additionally, the continued development of chimeric antigen receptor T-cell (CART) therapy could yield a novel, targeted approach to PBL treatment [20]. 

## Conclusions

While no standard of care exists for PBL and outcomes have historically been poor, particularly in those with advanced age, we demonstrate that daratumumab-mini-CHOP may be an effective and viable option in elderly patients who cannot tolerate more intensive regimens. This strategy maximizes the use of a plasma cell-targeted therapy with an attenuated anthracycline backbone.
